# Discovery and biosynthetic assessment of '*Streptomyces ortus'* sp. nov. isolated from a deep-sea sponge

**DOI:** 10.1099/mgen.0.000996

**Published:** 2023-05-11

**Authors:** Sam E. Williams, Catherine R. Back, Eleanor Best, Judith Mantell, James E. M. Stach, Tom A. Williams, Paul R. Race, Paul Curnow

**Affiliations:** ^1^​ School of Biochemistry, University of Bristol, University Walk, Bristol BS8 1TD, UK; ^2^​ Woolfson Bioimaging Facility, University of Bristol, University Walk, Bristol BS8 1TD, UK; ^3^​ School of Natural and Environmental Sciences, Newcastle University, King’s Road, Newcastle upon Tyne NE1 7RU, UK; ^4^​ School of Biological Sciences, University of Bristol, Tyndall Avenue, Bristol BS8 1TQ, UK

**Keywords:** antibacterial, antibiotics, comparative genomics, genome mining, marine actinomycetes, phylogenomics

## Abstract

The deep sea is known to host novel bacteria with the potential to produce a diverse array of undiscovered natural products. Thus, understanding these bacteria is of broad interest in ecology and could also underpin applied drug discovery, specifically in the area of antimicrobials. Here, we isolate a new strain of *Streptomyces* from the tissue of the deep-sea sponge *Polymastia corticata* collected at a depth of 1869 m from the Gramberg Seamount in the Atlantic Ocean. This strain, which was given the initial designation A15ISP2-DRY2^T^, has a genome size of 9.29 Mb with a G+C content of 70.83 mol%. Phylogenomics determined that A15ISP2-DRY2^T^ represents a novel species within the genus *Streptomyces* as part of the *Streptomyces aurantiacus* clade. The biosynthetic potential of A15ISP2-DRY2^T^ was assessed relative to other members of the *S *. *aurantiacus* clade via comparative gene cluster family (GCF) analysis. This revealed a clear congruent relationship between phylogeny and GCF content. A15ISP2-DRY2^T^ contains six unique GCFs absent elsewhere in the clade. Culture-based assays were used to demonstrate the antibacterial activity of A15ISP2-DRY2^T^ against two drug-resistant human pathogens. Thus, we determine A15ISP2-DRY2^T^ to be a novel bacterial species with considerable biosynthetic potential and propose the systematic name '*Streptomyces ortus'* sp. nov.

## Data Summary

This A15ISP2-DRY2^T^ whole-genome sequencing project has been deposited at GenBank/ENA/DDBJ under the accession number JAIFZO000000000. The version described in this paper is version JAIFZO010000000. The genome of A15ISP2-DRY2^T^ had six 16S rRNA genes, the sequences of which were submitted to the National Center for Biotechnology Information (NCBI) 16S rRNA database with the accession numbers ON356021–ON356026. The sequence of the partial cytochrome oxidase subunit I (COI)-encoding gene from *Polymastia corticata* was deposited in GenBank with the accession number OP036683.

**Impact Statement**

The genus *Streptomyces* has contributed more to our antibiotic arsenal than any other group of bacteria or fungi. Despite decades of exploration, global analysis has suggested that they still possess more undiscovered biosynthetic diversity than any other bacterial group. Therefore, isolating novel species of *Streptomyces* is a priority for antibiotic discovery. Here, we isolate a novel strain from a deep-sea sponge and use comparative cluster analysis to identify six biosynthetic clusters unique to our deep-sea strain. This work demonstrates the utility of continuing to isolate novel *Streptomyces* strains for antibiotic discovery and, for the first time, we used species tree–gene cluster tree reconciliation to assess the contribution of vertical evolution on the biosynthetic gene cluster content of *Streptomyces *.

## Introduction

Antimicrobial resistance is a major threat to human health and was associated with nearly five million deaths worldwide in 2019 [1]. The discovery of new antibiotics with novel modes of action is a critical part of combatting this threat. In recent years, there has been a renewed focus on microbial natural products as the basis for this discovery [2]. Historically, the majority of antibiotic natural products have come from the bacterial group actinomycetes, with the majority of these arising from a select few genera such as *Streptomyces* and *Micromonospora* [3, 4]. It has been estimated that over 50 % of all clinically used antibiotics are derived from the *Streptomycetes* alone [4–7].

While *Streptomyces* remain an attractive target for biodiscovery initiatives, these efforts can be generally frustrated by the continual re-discovery of known natural products [8]. One way to mitigate this problem is to focus upon relatively under-sampled environmental niches that could harbour strains which have acquired biosynthetic innovations [9, 10]. The microbial fauna intimately associated with marine sponges have long been seen as a potential source of novel bioactive metabolites, and *Streptomyces* strains are known to feature in sponge microbiota [11, 12]. While the bioprospecting of sponge samples has largely been limited to accessible shallow waters [13], a more ‘extreme’ niche occupied by sponges – the deep ocean – has been less well explored [14]. It is now apparent that deep-sea sponges can indeed host microbial communities with impressive bioactivity, and that many such communities include the *Streptomycetes* [15–17].

As well as traditional culture-based methods, bioinformatic genome mining for biosynthetic gene clusters (BGCs) now has a central role in the process of natural product discovery [5, 18]. The identification of biosynthetic clusters by tools such as antiSMASH [19] is complemented by software that can, for example, predict whether a particular gene cluster might produce an antibiotic [20]. Grouping together similar BGCs from multiple genomes into gene cluster families (GCFs) has provided a deeper understanding of global biosynthetic diversity [21–23] and has been used to estimate that just 3 % of bacterial natural product classes have so far been discovered [24]. In such analyses, *Streptomyces* are found to have the greatest biosynthetic potential, with certain phylogenetic subgroupings within the genus – specifically, a clade termed ‘* Streptomyces* _RG1’ (relative evolutionary distance or RED group 1) – expected to be biosynthetically exceptional even by the standards of other *Streptomyces* [24]. The isolation, identification and characterization of novel taxa within this ‘RG1’ clade is, therefore, a priority for natural product discovery.

Here, we describe the discovery of a novel species of deep-sea *Streptomyces* with a diverse biosynthetic repertoire and inherent antibiotic activity. We investigate the biosynthetic potential of this strain through a comparative analysis of GCF diversity within the *Streptomyces aurantiacus* clade [25] and highlight the extent that specialized metabolism within this clade remains unexplored.

## Methods

### Sponge identification

The sponge sample was thoroughly rinsed three times in sterile artificial seawater (33.3 g Crystal Sea Marine Mix l^–1^; Marine Enterprise International). DNA was extracted from ~0.25 g sponge tissue in a laminar flow hood using the DNeasy PowerSoil kit (Qiagen) using the optimized procedure of Marotz *et al*. [26]. Sponge taxonomy was based on the mitochondrial cytochrome oxidase subunit I (COI). The COI-encoding gene was amplified through PCR using the universal primer pair LCO1490 (5'-GGTCAACAAATCATAAAGATATTGG-3') and HCO2198 (5'-TAAACTTCAGGGTGACCAAAAAATCA-3') [27]. The reaction comprised 20 µl Platinum Hot Start PCR master mix (Thermo Fisher Scientific), 2 µl each primer at 10 pmol µl^−1^, 14 µl MilliQ water and 2 µl (130 ng/µl) DNA template. Thermocycler conditions were as described by Yang *et al*. [28]: 1 min denaturation at 94 °C; 5 cycles of 94 °C for 30 s, 45 °C for 90 s and 72 °C for 1 min; 35 cycles of 94 °C for 30 s, 51 °C for 40 s and 72 °C for 1 min; and a final extension step at 72 °C for 5 min. The successful amplification of a COI-encoding gene fragment of approximately 680 bp was confirmed by agarose gel electrophoresis before the amplicon was purified with a DNA Cleanup kit (New England Biolabs) and sequenced (Eurofins Genomics). The closest relative was determined based on the highest per cent identity in a blastn search against the NCBI Nucleotide database [29].

### Isolation of A15ISP2-DRY2^T^

The procedures for strain culturing and isolation, and the method of antibiotic screening by soft agar overlay, were performed as previously described [30]. To bias strain culturing towards spore-forming bacteria, a dry-stamping technique was adapted from Mincer *et al.* [31]. A sample of 0.25 g sponge was dried at 60 °C for 90 min, ground with a sterile pestle, and then stamped onto ISP2 (4.0 g glucose, 4.0 g yeast extract, 10.0 g malt extract, 33.3 g Crystal Sea Marine Mix, 15 g agar, 1 l ddH_2_O) agar plates using a sterile plastic bung.

### Cryo-scanning electron microscopy (cryo-SEM) imaging

A15ISP2-DRY2^T^ was streaked onto mannitol soya flour medium [32], grown for 4 days at 28 °C and a single colony was removed with a scalpel. For SEM, colonies were mounted on the surface of an aluminium stub with optimal cutting temperature (OCT) compound (Agar Scientific) mixed with colloidal graphite as the mounting medium. The stub was plunged into liquid nitrogen slush to cryopreserve the material. Each sample was transferred to the preparation chamber of a Quorum PP3010T cryo-transfer system attached to a JEOL 7900 field emission scanning electron microscope. Sublimation of surface frost was performed at −95 °C for 3 min before re-cooling then sputter coating with platinum for 2 min at 10 mA. After coating, the sample was transferred to the cryo stage mounted in the SEM chamber held at approximately −140 °C. The samples were imaged at 5 kV. Mean spore dimensions were determined with Fiji ImageJ v2.3.0 using the UCSB NanoFab plugin Microscope Measurement Tools package.

### Strain growth conditions

The standard growth conditions for culturing A15ISP2-DRY2^T^ were on standard ISP2 media (4.0 g glucose, 4.0 g yeast extract, 10.0 g malt extract, 15 g agar, 1 l ddH_2_O) at 28 °C, pH 7.2. To examine the impact of different growth conditions, strains were cultured in triplicate on ISP2 agar over a range of temperatures, salinities and pH values deviating from the standard culture conditions. Growth temperatures on standard ISP2 were 0, 4, 15, 20, 28 and 37 °C. Additional NaCl was introduced at concentrations between 0, 2, 4, 6, 8, 10, 12 and 15 % (w/v). pH was adjusted to final values of 5, 6, 7, 8, 9, 10, 11 and 12 with 2 M HCl or 2 M NaOH. Plates incubated at 0 °C were also supplemented with 3 % (v/v) glycerol to prevent freezing. Gram staining was carried out as described elsewhere [33].

### Analysis of fatty acid cell wall composition

The chemotaxonomic analysis sample was prepared from biomass produced in M.65 medium (L-1) containing 4.0 g glucose, 4.0 g yeast extract and 10.0 g malt extract (pH 7.2). Whole-cell sugars and isomers of diaminopimelic acid were diagnosed with standard samples by TLC on cellulose plates according to the method of Staneck and Roberts [34]. Polar lipids were extracted from freeze-dried material in chloroform:methanol:0.3 % aqueous NaCl, separated by 2D TLC and detected according to the study by Tindall and colleagues [35]. Cellular fatty acids were extracted, methylated and analysed using minor modifications of the methods of Miller [36] and Kuykendall *et al*. [37]. The fatty-acid methyl esters were separated by GC and identified using the Sherlock Microbial Identification System (MIDI). Menaquinones were extracted in hexane, purified by silica-based solid phase extraction and analysed by reverse phase HPLC-DAD-MS. Phenotypic characterization was done in BioLog GENIII MicroPlates (71 carbon source utilization assays, 23 chemical sensitivity assays) with further biochemical tests done with API 20E strips. All analyses were performed by DSMZ services, Leibniz-Institute DSMZ, Braunschweig, Germany.

### Genome assembly

A colony was inoculated from a freshly streaked ISP2 agar plate into 1 ml liquid media of the same composition. These liquid cultures were incubated in a shaking incubator (180 r.p.m., 28 °C) for 3–4 days until confluent bacterial growth was achieved. Genome extraction was then performed using the GenElute bacterial genomic DNA extraction kit (Sigma-Aldrich), as per the manufacturer’s instructions. Illumina sequencing was performed as a commercial service by Microbes NG (https://microbesng.com/). Briefly, libraries were constructed using the XT Index kit (Nextera) and sequenced using HiSeq or NovoSeq platform (Illumina) to produce 2×250 bp paired-end reads. Trimmomatic (v0.30) was used for adaptor and quality trimming with a sliding window quality cut-off of Q15 and a minimum read length of 36 bp [38]. Trimmomatic quality trimming resulted in 70 298 read pairs being removed from a total of 1 627 094. Nanopore sequencing was conducted in-house. Extracted genomic DNA was sequenced using the rapid sequencing kit (SQK-RAD004) on an Mk1B R9 MinION flow-cell (Oxford Nanopore Technologies) and raw fast5 files were base called using Guppy (v6.3.8). Sequencing files were assembled *de novo* with Unicycler v0.4.6 [39] and the assembly was scaffolded with MeDuSa v1.6 [40] (reference strains are listed in Table S1). Alignment of trimmed Illumina reads to the final assemblies used Bowtie2 v2.2.9 [41], error rate and coverage of Illumina reads across the assembly were calculated with Qualimap2 v2.2.2 (Table S2, Fig. S1) [42]. The contiguity and accuracy of the assembly were assessed, respectively, with quast v5.0.2 [43] and Benchmarking Universal Single-Copy Orthologs (BUSCO) (v5.3.2) [44]. Assembly and quality assessment were completed on the Galaxy web platform (https://usegalaxy.eu/) [45].

### Phylogenomics

The final genome assembly file was submitted to the DSMZ Type Strain Genome Server (TYGS v.321; https://tygs.dsmz.de/) and the Genome to Genome Distance Calculator (GGDC v2.1; https://ggdc.dsmz.de) [46]. Phylogenetic trees produced by TYGS were visualized using the interactive tree of life (iTOL) V5 [47] with *Micromonospora echinospora* ATCC 15837 selected as an outgroup. Average nucleotide identity (ANI) was calculated on the Galaxy web platform (https://usegalaxy.eu/) using the FastANI algorithm [48] against the closest relatives identified by TYGS. The genome was submitted for annotation to the NCBI Prokaryote Genome Annotation Pipeline (PGAP) (v6.3) [49].

### Biosynthetic cluster comparison of the *S. aurantiacus* clade

BGCs from the *S. aurantiacus* clade and the isolated strain were identified using antiSMASH 6.1 [19]. Where multiple assemblies were available, the highest-quality genome for each species in GenBank was chosen (Table S1). To enable cluster comparison, detected BGCs were grouped into GCFs using BiG-SCAPE v1.1.0 [21]. BiG-SCAPE was run with the *--mix* flag and the clustering distance parameter was tested at 0.3, 0.35, 0.4, 0.45 and 0.5; 0.35 was chosen, as this represented the lowest cut-off that grouped the geosmin BGC into a single GCF [50]. The resulting network map output was loaded into Cytoscape v3.9.1 [51]. The *--mibig* flag was used to identify closely related clusters from the Minimum Information about a Biosynthetic Gene cluster (MIBiG 2.0) database [52]. For each GCF, aligned amino acid sequences produced by BiG-SCAPE were used as input for iq-tree2 [53] to create 1000 bootstrapped GCF trees using the best-fitting substitution model as selected by the Bayesian Information Criterion. Bootstrapped trees were used as input for ALEobserve and then reconciled against the rooted species tree using ALEml_undated [ALE (Amalgamated Likelihood Estimation) v1.0] [54]. The verticality of each branch was then calculated as the inferred number of ancestor-to-descendant vertical transmissions as a proportion of all inferred events.

### Bioactivity testing

A cube of mycelium containing agar was used to inoculate a starter culture of 50 ml liquid ISP2 without glucose in 250 ml baffled Erlenmeyer flasks. After 24 h growth at 28 °C with agitation at 180 r.p.m., 10 ml of the starter culture was used to inoculate 100 ml ISP2 media in 250 ml baffled Erlenmeyer flasks and grown for 7 days (28 °C, 180 r.p.m.). The total culture was then extracted through rigorous shaking with an equal volume of ethyl acetate (Sigma-Aldrich). The organic layer was removed and dried over anhydrous MgSO_4_ (Sigma-Aldrich) before being evaporated under vacuum. The dried crude extract was resuspended in 2 ml methanol for bioactivity testing.

Bioactivity testing employed the following ESKAPE pathogens: *Staphylococcus aureus* Mu50, *Klebsiella pneumoniae* NCTC 5055, *Acinetobacter baumannii* ATCC 19606, *Pseudomonas aeruginosa* PAO1, *Escherichia coli* BW55113 and *Enterococcus faecalis* UB591 (JH2-2). All strains were grown in Muller–Hinton broth at 37 °C for 16 h with shaking at 180 r.p.m. Cultures were diluted to OD_600_0.01 in warm, molten Muller–Hinton agar (0.75 % agar), and 10 ml was poured over a plate of solid Muller–Hinton agar and allowed to set. The crude extract of A15ISP2-DRY2^T^ (25 µl) was dried onto sterile discs of filter paper, which were placed onto the surface of the set agar. ISP2 media extracts were used as a negative control and discs containing levofloxacin (5 µg) were used as positive controls. The plates were incubated at 37 °C, for 16 h, at which point zones of inhibition (ZOIs) could be observed.

## Results

### Isolation of strain A15ISP2-DRY2^T^

**Fig. 1.**
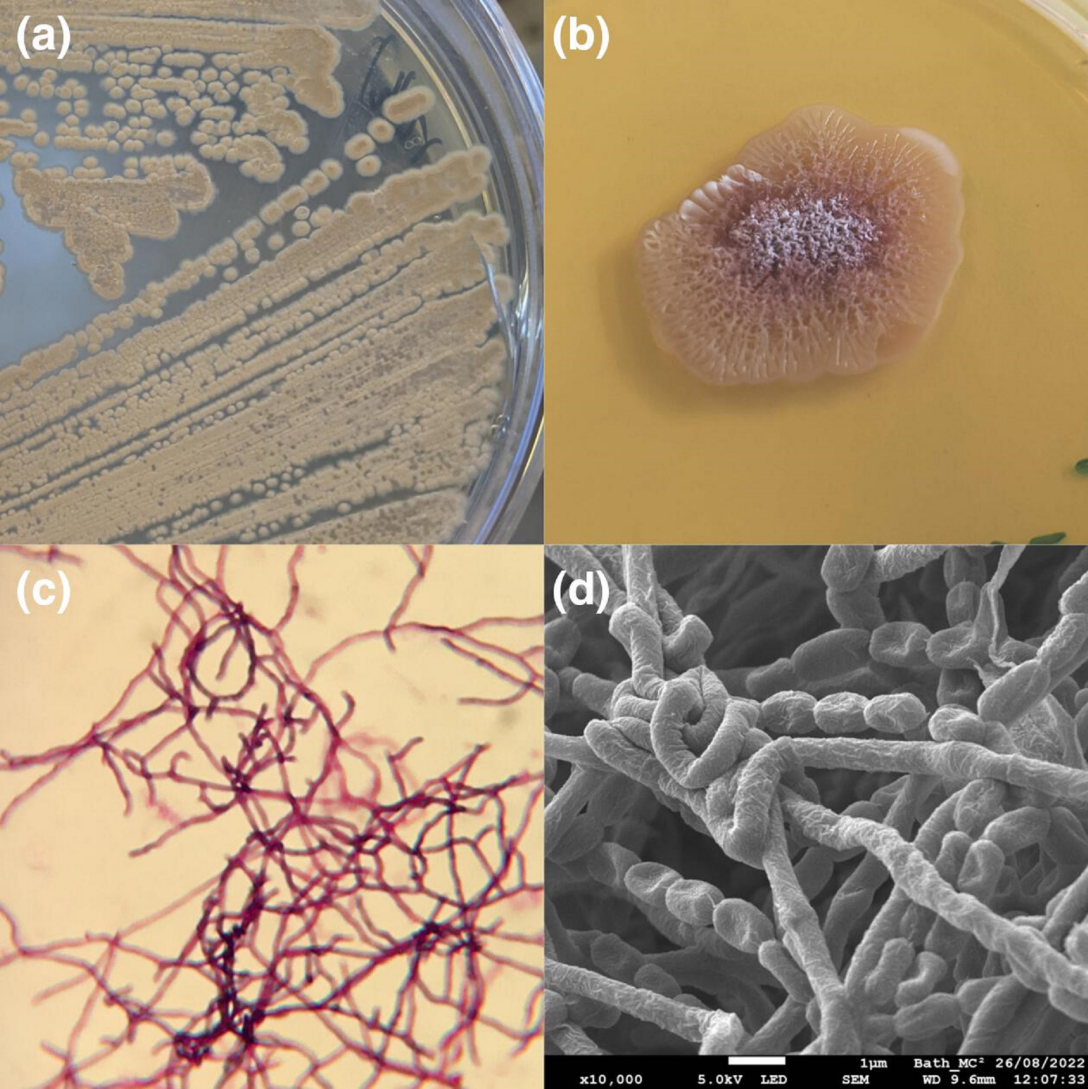
Images of microbial isolate A15ISP2-DRY2 ^T^. (**a**) Colonies streaked and growing on ISP2 agar. (**b**) A large colony after 1 week of growth, colonies were initially cream but over time changed to orange and finally burgundy. White spores can be seen on the colony (**c**) The strain was Gram-positive and showed filamentous structures when viewed under a light microscope. (**d**) Cryo-SEM micrographs of A15ISP2-DRY2 ^T^ colony grown on mannitol soya flour medium for 4 days.

A bacterial strain given the in-house designation A15ISP2-DRY2^T^ was isolated on ISP2 agar as part of an ongoing effort to culture bacteria from the microbiota of deep-sea sponges [30]. The sponge sample was originally recovered by remote-operated vehicle from the Gramberg Seamount in the Atlantic Ocean (depth 1869 m; latitude 15° 26' 43″ N; longitude 51° 05' 46″ W). This was identified as the demosponge *Polymastia corticata* based on cytochrome oxidase I (COI)-encoding gene identity (Table S3; GenBank accession no. OP036683). Strain A15ISP-DRY2^T^ produced a ZOI against the Gram-positive test strain *Bacillus subtilis* in a soft agar overlay assay, but did not inhibit the growth of the Gram-negative test strain *Escherichia coli* (data not shown). A15ISP2-DRY2 ^T^ was shown to be Gram-positive, and electron microscopy of the strain revealed branching filamentous substrate and spores (Fig. 1).

### Taxonomic assignment

**Fig. 2.**
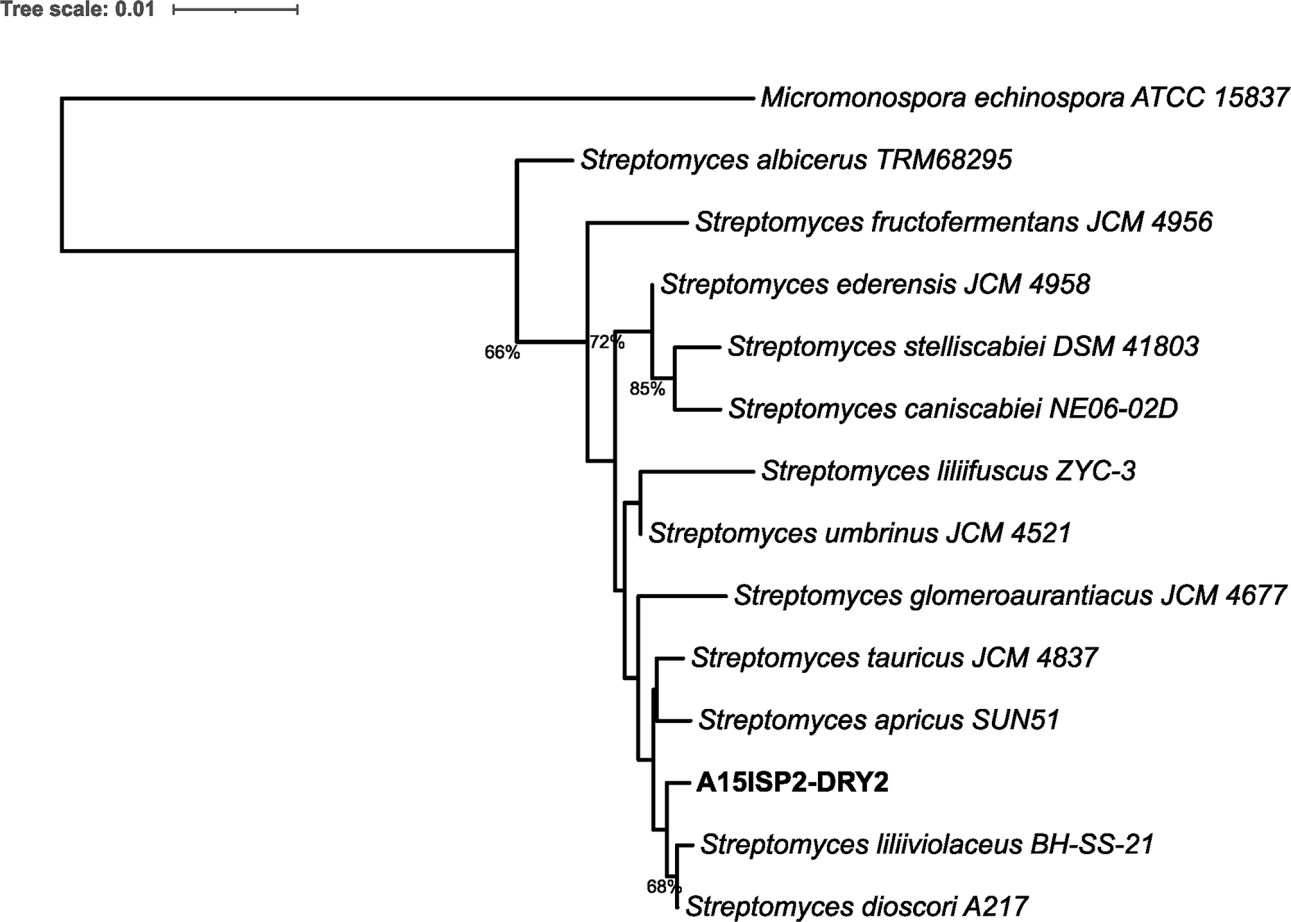
16S rRNA gene sequence phylogeny of A15ISP-DRY2^T^. Produced with TYGS, bootstrap values below 50 % not shown, mean branch support 49.5%, 14 strains, δ statistics=0.455. The branch lengths are scaled in terms of Genome BLAST Distance Phylogeny (GBDP) distance formula d_5_. *M. echinospora* ATCC 15837 is used as an outgroup and the tree is rooted on this branch.

A full-length 16S rRNA gene sequence of 1525 bp (GenBank accession no. ON356025.1) was sequenced and submitted to NCBI blastn 2.13.0+. A phylogenetic analysis of closely related type strains indicated that the deep-sea isolate was a member of the *S. aurantiacus* clade [25] (Fig. 2). Within this clade, the closest relatives to the isolate were the strains '*Streptomyces dioscori'* A217^T^ and *Streptomyces liliiviolaceus* BH-SS-21^T^ with 16S rRNA gene identities of 99.74 and 99.61 %, respectively.

The phenotypic properties of strain A15ISP2-DRY2^T^ are given in Table 1. Major fatty acids (>10 %) for strain A15ISP2-DRY2^T^ were anteiso-C_15 : 0_, C_16 : 0_, iso-C_16 : 0_ and C_16 : 1_ *ω*7*c*. Minor fatty acids (>5 %) were iso-C_14 : 0_, iso-C_15 : 0_ and anteiso-C_17 : 0_. Whole-cell sugars detected were glucose, galactose, ribose and minor amounts of mannose. The diagnostic amino acid in whole-cell hydrolysates was ll-2,6-diaminopimelic acid. Menaquinones MK8 H6 (10.5 %), MK8 H8 (0.8%), MK9 H4 (8.1 %), MK9 H6 (50.5 %) and MK9 H8 (30.1 %) were present.

**Table 1.** Physiological characteristics of strain A15ISP2-DRY2^T^ All analyses for strain A15ISP2-DRY2 were performed at DSMZ services, Leibniz-Institute DSMZ, Germany.

Characteristic	A15ISP2-DRY2^T^
**Growth on sole carbon sources:**	
d-Fructose	+
l-Arginine	−
l-Arabinose	−
Inositol	−
Sodium citrate	−
**Hydrolysis of:**	
Gelatine	+
Tween 40	+
**Activity:**	
H_2_S production	−
Voges–Proskauer	+
**Major fatty acids:**	
Iso-C_17 : 1_ *ω*5*c*	−
**Polar lipids:**	
Phosphatidylinositol	+
Phosphatidylinositol mannoside	−

The genome of A15ISP2-DRY2^T^ was sequenced to allow complete taxonomic assignment and assess the biosynthetic potential of this strain. The complete assembled genome was 9 291 524 bp in length, with a G+C content of 70.83 mol%. The assembly consisted of nine contigs, or four scaffolds, with an L50 of one and the largest scaffold being 8 605 295 bp in length (Table S2). The assembled genome contained 8130 genes, 6 complete rRNAs, 66 tRNAs and a single CRISPR array (Table S4). The genome was further analysed for expected single-copy orthologous genes from the order *Streptomycetales *. Of 1579 expected genes, 1574 were complete (99.7 %), suggesting the assembly was of high biological accuracy (Table S5).

**Fig. 3.**
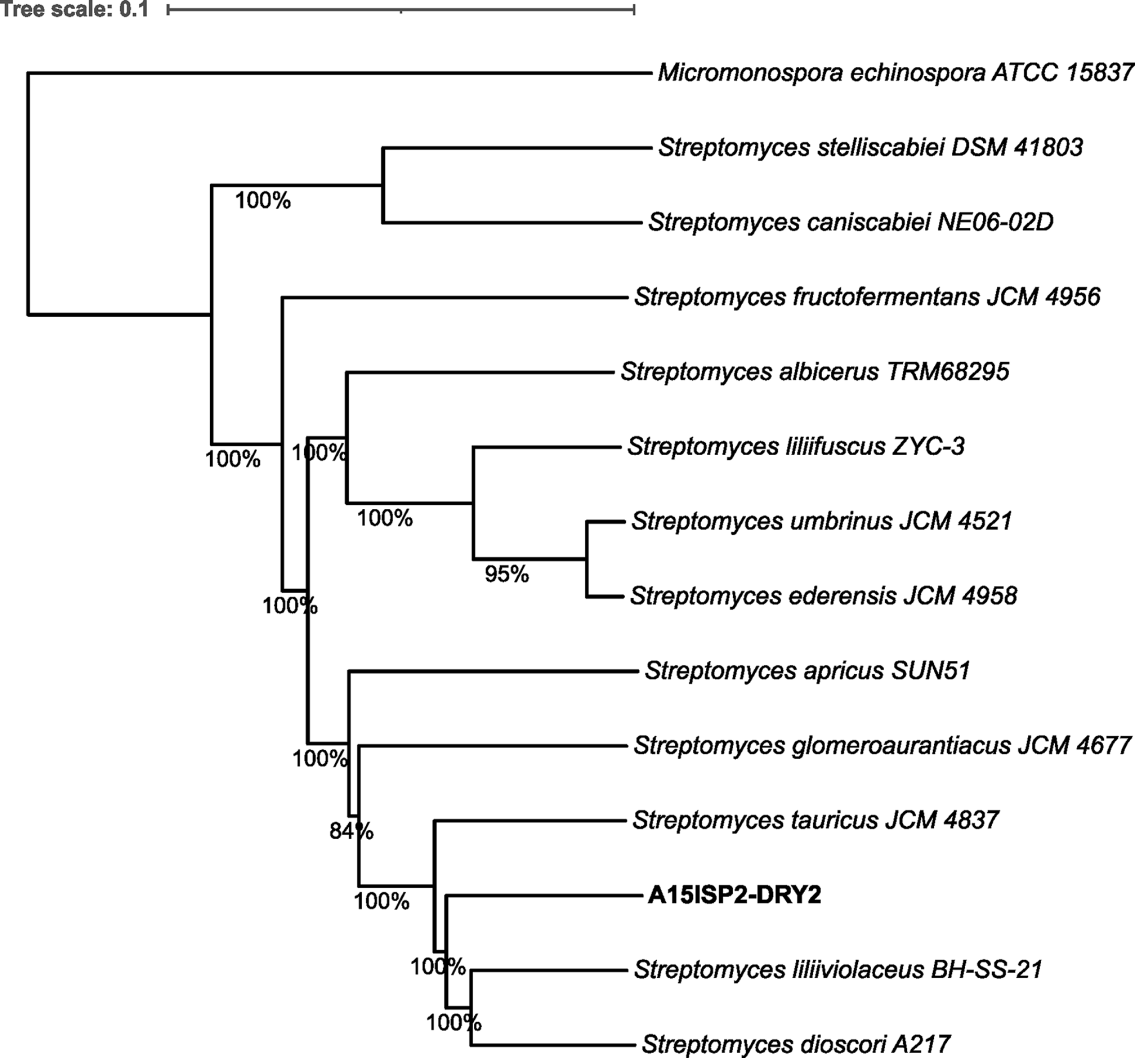
Whole-genome phylogenetic tree produced with TYGS . Thirteen strains were automatically chosen using TYGS with GGDC. *M. echinospora* ATCC 15837 is included as an outgroup and the tree is rooted on this branch. Mean branch support 98.1 %, bootstrap data shown as a per cent for each branch. δ statistics=0.131. Excluding the outgroup: G+C 70.02–72.41 mol%, genome size 7.67–11.96 Mb, number of proteins 6307–10 784. The branch lengths are scaled in terms of GBDP distance formula d_5_.

TYGS (https://tygs.dsmz.de) [46] was used to calculate digital DNA–DNA hybridization (dDDH) values and to create a whole-genome phylogeny (Fig. 3). FastANI was then used to report the ANI between the isolated strain and closely related type strains [55] (Table S6). This was consistent with the results of 16S analysis and confirmed that the closest relatives to A15ISP2-DRY2^T^ are *S. liliiviolaceus* BH-SS-21^T^, with dDDH score of 45.8 %, ANI 93.31 % and G+C difference 0.04 mol%, and *'S. dioscori'* A217^T^, with dDDH 45.1 %, ANI 93.08 % and G+C difference 0.11 mol%. The relative dissimilarity of the dDDH and ANI scores between A15ISP2-DRY2^T^ and these related strains reveals that A15ISP2-DRY2^T^ should be considered a distinct species. Thus, we propose here that A15ISP2-DRY2^T^ be given the systematic name '*Streptomyces ortus'* sp. nov. A species description, including an explanation of the epithet and details of culture deposition in accessible collections, is provided at the end of this article.

The whole-genome analysis confirmed the placing of A15ISP2-DRY^T^ within the *S. aurantiacus* clade. This is of interest since the *S. aurantiacus* clade is part of a within-taxon group known as *Streptomyces_RG1* that was recently assigned based on relative evolutionary distance (RED) [24]. *Streptomyces_RG1* are expected to have the highest biosynthetic potential of any genus-level group in the bacterial kingdom. This suggests that the *Streptomyces* isolate described here represents an excellent candidate for further bioprospecting.

### Analysis of BGCs

The genome of A15ISP2-DRY2^T^ was analysed with antiSMASH 6.1.1 for the identification of putative BGCs. A total of 34 complete BGCs were identified. Just nine of these showed high gene similarity (>80 %) to currently characterized BGCs (Table 2). Only 19 of the identified BGCs were most similar to those found in the closest relative listed in the antiSMASH database *'S. dioscori'* A217 (Table S7). Additionally, the genome was submitted to Antibiotic Resistant Target Seeker 2 (ARTS; https://arts.ziemertlab.com) [56]. This identified that 19 of the 34 identified BGCs in A15ISP2-DRY2^T^ were in proximity to a duplicated core gene or a known antibiotic-resistance gene. This genomic context indicates that these BGCs may produce antibiotic compounds [18, 57].

**Table 2.** Output from antiSMASH 6.1.1 for the genome assembly of isolate A15ISP2-DRY2^T^

Region	Type	Cluster size (bp)	Most similar known cluster	Similarity
1	NRPS, T3PKS	117 822	Herboxidiene	10 %
2	Terpene	21 059	2-Methylisoborneol	100 %
3	NRPS	56 791	Foxicins A–D	29 %
4	Siderophore	13 852	*No similar cluster*	–
5	NAPAA	33 969	Rapamycin	17 %
6	Ectoine	10 405	Ectoine	100 %
7	Terpene	21 086	Albaflavenone	100 %
8	PKS-like, T1PKS	48 134	Arsono-polyketide	91 %
9	T3PKS	41 119	Alkylresorcinol	66 %
10	NRPS, T3PKS, terpene	122 195	Feglymycin	73 %
11	T2PKS, ladderane	76 451	Simocyclinone D8	40 %
12	Terpene	25 548	Isorenieratene	100 %
13	NRPS	77 342	Borrelidin	4 %
14	NRPS	49 245	Rimosamide	21 %
15	NRPS	44 350	Diisonitrile antibiotic SF2768	55 %
16	Lanthipeptide – class-iii, RiPP-like	10 216	Informatipeptin	42 %
17	T1PKS, terpene	42 550	Oxalomycin B	9 %
18	Terpene	19 424	Herboxidiene	4 %
19	NRPS, NRPS-like, T1PKS, other, terpene	187 132	Aurantimycin A	48 %
20	Terpene	24 515	Hopene	92 %
21	NRPS-like, PKS-like, T1PKS, ectoine	52 830	Showdomycin	17 %
22	Siderophore	11 933	Grincamycin	8 %
23	NAPAA	34 968	Stenothricin	13 %
24	Terpene	22 187	Geosmin	100 %
25	RiPP-like	10 685	*No similar cluster*	–
26	NRPS, NRPS-like, β-lactone	91 586	Vazabitide A	23 %
27	Lanthipeptide class iv	22 754	*No similar cluster*	–
28	Siderphore	13 568	*No similar cluster*	–
29	PKS-like, RRE-containing, T2PKS	72 534	Cinerubin B	100 %
30	Melanin	10 516	Melanin	60 %
31	Siderophore	11 788	Desferrioxamine B/E	83 %
32	RiPP-like	11 875	*No similar cluster*	–
33	Nucleoside	20 705	*No similar cluster*	–
34	NRPS	44 035	Lysolipin I	4 %

### Biosynthetic cluster comparison within the *S. aurantiacus* clade

**Fig. 4.**
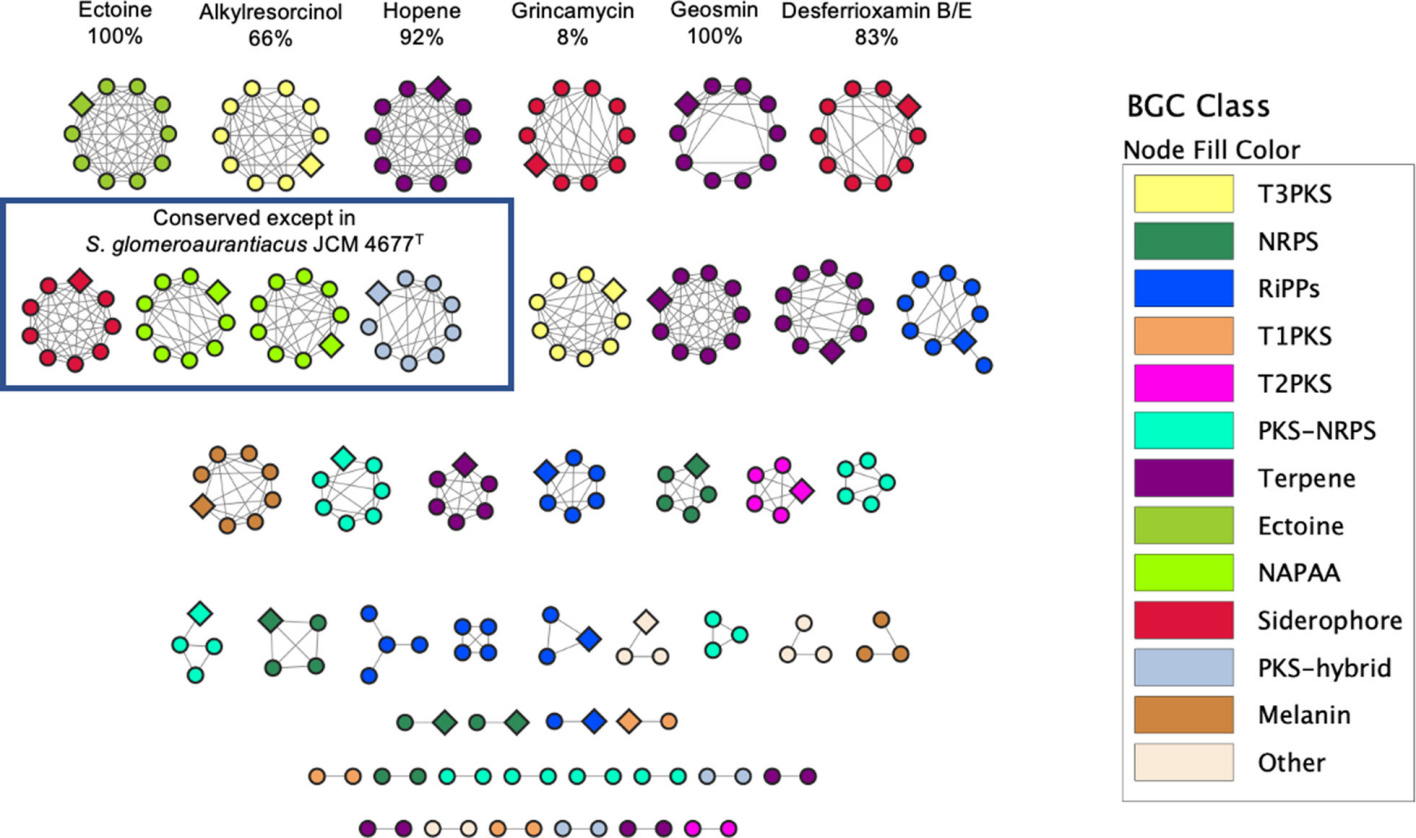
BiG-SCAPE GCF network analysis of 10 *Streptomyces* within the *S. aurantiacus* clade, including A15ISP2-DRY2^T^. Conserved GCFs are annotated with the name of the compound produced by the most similar known cluster, and antiSMASH percent gene similarity to that cluster. Diamond nodes are BGCs belonging to A15ISP2-DRY2^T^. Fragmented BGC nodes were removed so that only one node per GCF per strain was left in the network. Singletons are excluded from the figure. The key shows compounds produced by gene products of the most similar known cluster. NRPS, Non-ribosomal polyketide synthase, here including NRPS-like clusters; RiPP, ribosomally synthesized and post-translationally modified peptides, here including lanthipeptides and RiPP-like clusters; NAPAA, non-alpha poly-amino acids; PKS-hybrid, here including T1PKS-terpene, heterocyst glycolipid synthase-like PKS-T1PKS, Other, here including redox-cofactor, phosphoglycolipid and nucleoside; T1PKS, type I polyketide synthase; T2PKS, type II polyketide synthase; T3PKS, type III polyketide synthase.

To further evaluate the biosynthetic novelty of A15ISP2-DRY2^T^, a BiG-SCAPE GCF analysis [21] was conducted within a clade of the 10 closest relatives. A total of 377 BGCs were identified across the 10 genomes analysed. These grouped into 188 GCFs with 133 singletons and a total of 577 links (Figs 4 and S2). Just 22 (11.7 %) of these GCFs clustered with a MIBiG reference BGC [52], highlighting the extent of unexplored specialized metabolism within this clade. Just six biosynthetic GCFs were shared amongst all members of the clade (Fig. 4). Less stringently, ten GCFs were near-ubiquitous, being conserved in all members of the clade apart from *Streptomyces glomeroaurantiacus* JCM 4677^T^. The six conserved GCFs included the well-characterized clusters responsible for producing ectoine, hopene, geosmin, desferrioxamine B/E and alkylresorcinol, which are common across most *Streptomyces* strains [58]. The other GCF found ubiquitously across the clade was a siderophore cluster with low gene similarity (8 %) to that for the antibiotic polyketide grincamycin [59].

The phylogenetic subgroup encompassing A15ISP2-DRY2^T^, *S. liliiviolaceus* BH-SS-21^T^, *'S. dioscori*' A217 and *Streptomyces tauricus* JCM 4837^T^ exclusively contained GCFs for a polyketide synthase non-ribosomal polyketide synthase (PKS-NRPS) hybrid cluster and an NRPS cluster. The subgroup also contained the type II polyketide synthase (T2PKS) for the anthracycline cinerubin B [60]. Interestingly the cinerubin B GCF was also found in *Streptomyces fructofermentans *, suggesting this GCF has moved via lateral gene transfer (LGT) into the clade on two separate events. A nucleoside GCF with no similar other known cluster was found in all members of the group except *S. tauricus *. A15ISP2-DRY2^T^ and its closest relative *S. liliiviolaceus* BH-SS-21^T^ also had two GCFs not found elsewhere, including a NRPS-like cluster with 23 % similarity to a cluster for vazabitide A [61]. This BGC was deemed likely to produce an antimicrobial, with ARTS 2.0 identifying a duplicated core gene and a putative resistance model within the cluster. Overall, this analysis demonstrates a clear phylogenetic relationship of BGC distribution in this clade, with close relatives sharing a higher portion of conserved BGCs.

A15ISP2-DRY2^T^ had six GCFs not found in any other members of the clade, potentially suggesting six BGC acquisition events since its speciation from *S. liliiviolaceus *. Two of these unique GCFs are likely to produce antibiotics based on ARTS analysis. These are a T2PKS and ladderane hybrid BGC, containing a known resistance model and 40 % gene homology to the novel DNA gyrase inhibitor simocyclinone D8 [62]; and an NRPS BGC with a duplicated cell envelope core gene within the cluster. A15ISP2-DRY2^T^ also had a unique large NRPS-type III polyketide synthase (T3PKS) hybrid BGC with 73 % similarity to the glycopeptide-related antibiotic feglymycin, but ARTS did not detect any resistance markers for this cluster. Thus, there appear to be uncharacterized BGCs within A15ISP2-DRY2^T^, responsible for producing antibiotics and not found in closely related *Streptomyces* species.

### Predominantly vertical GCF transmission within the *S. aurantiacus* clade

**Fig. 5.**
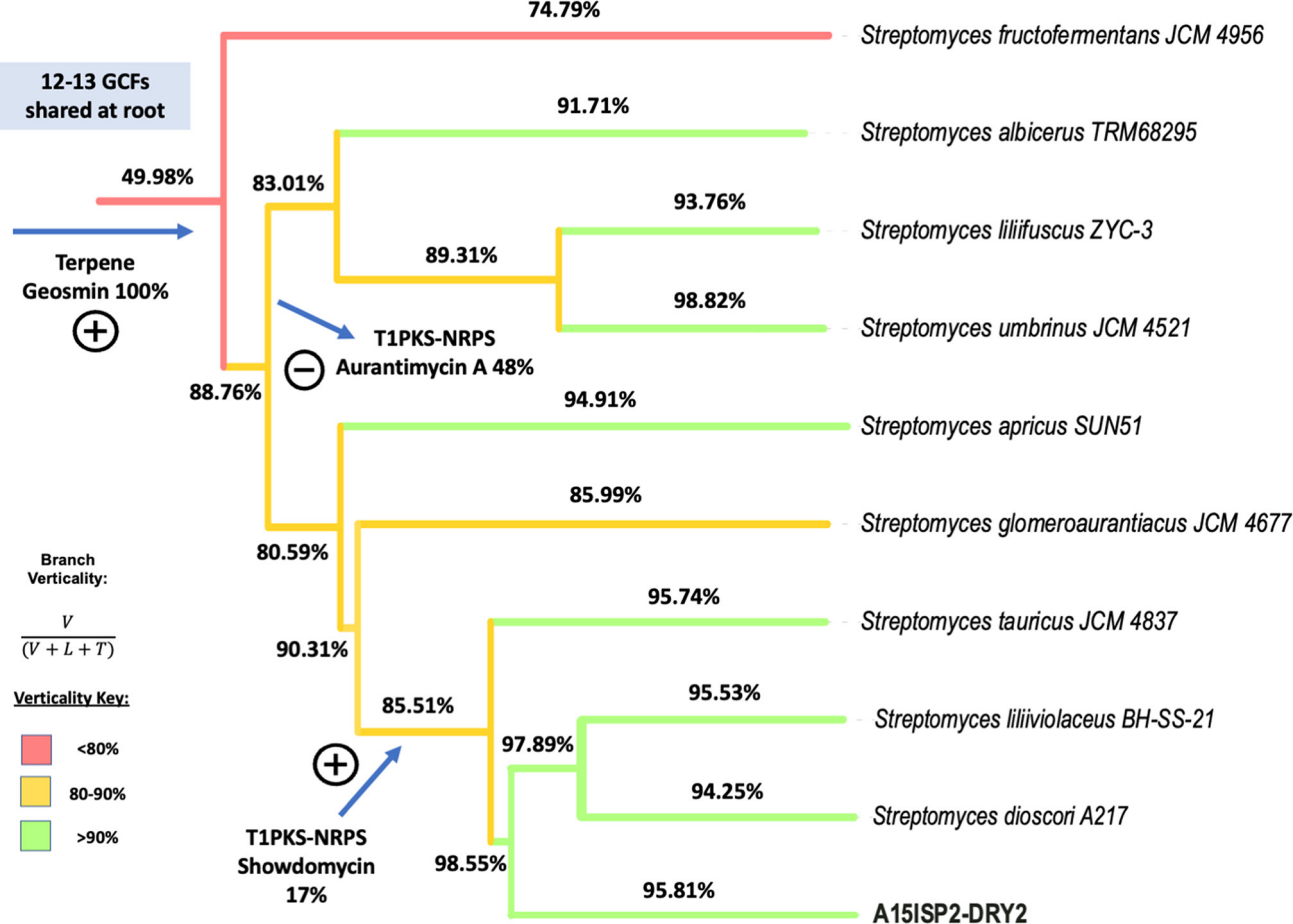
GCF transmission within the *S. aurantiacus* clade revealed using gene cluster tree–species tree reconciliation. ALE analysis showed the evolution of 28 GCFs present in three or more strains. These clusters generally evolved vertically, with >90 % verticality on most branches of the tree (verticality was measured as the proportion of ancestor-to-descendant vertical transmissions as a proportion of all inferred events). For illustrative purposes, the inferred origin or loss of three clusters is illustrated. *V*=Number of vertical transmissions; *L*=number of loss events; *T*=number of transfers.

To investigate the contributions of vertical inheritance and LGT to the evolution of BGCs within the *S. aurantiacus* clade, we performed gene cluster tree–species tree reconciliation. In total, there were 28 GCF trees, where the GCF was present in at least three strains, which were reconciled against the clade species tree using ALE [54]. ALE draws the gene tree into the species tree using a probabilistic model of gene origination, duplication, transfer and loss, with model parameters estimated from the data using maximum likelihood. Consistent with the observed congruence between the species tree and BGC repertoires of these organisms, the ALE analysis suggested that vertical inheritance was the predominant mode of BGC evolution within the *S. aurantiacus* clade. The GCF branch verticality, a measure of vertical transmission in relation to transfer or loss, demonstrated a mean GCF verticality of 89 %. This analysis suggested that 12 to 13 of the GCFs analysed were already present in the last common ancestor of the *S. aurantiacus* clade (Fig. 5). The analysis also highlighted loss events, such as a type I polyketide synthase (T1PKS)-NRPS BGC with homology to aurantimycin A, and evidenced acquisition events, such as a T1PKS-NRPS BGC with low homology to showdomycin in the '*S. ortus'* group.

### Antimicrobial-activity testing

**Fig. 6.**
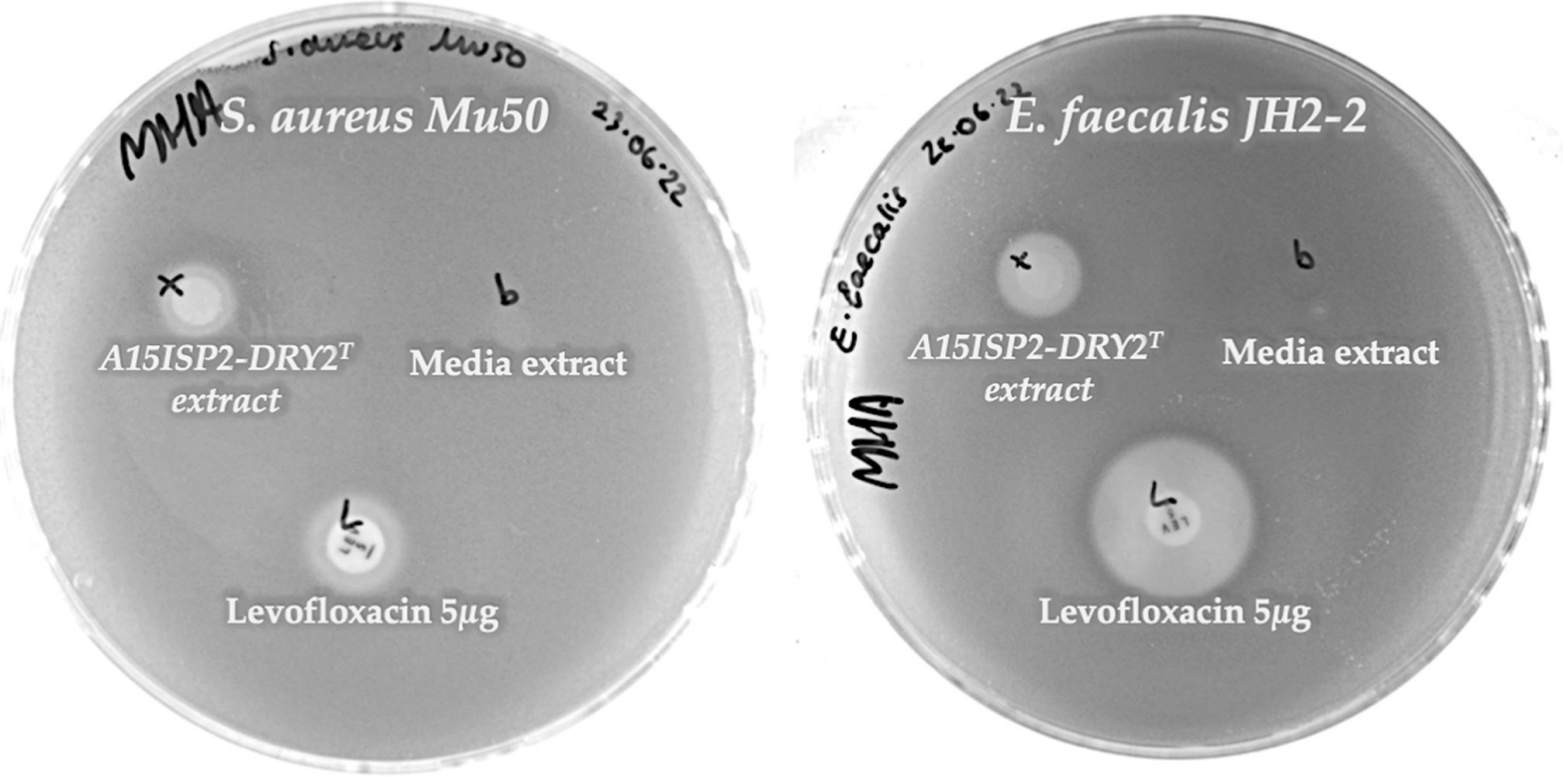
Example of antibiotic activity in crude media extracts of A15ISP2-DRY2^T^. ZOIs are visible in agar overlay assays against the pathogens: *Staphylococcus aureus* Mu50 (left) – A15ISP2-DRY2 ^T^ ZOI diameter=10.3 mm, levofloxacin ZOI diameter=14.3 mm; and *Enterococcus faecalis* JH2-2 (right) – A15ISP2-DRY2 ^T^ ZOI diameter=12.2 mm, levofloxacin ZOI diameter=23.5 mm. The broad-spectrum antibiotic lexofloxacin is used as a positive control.

Following the bioinformatic analysis, we next tested whether this A15ISP2-DRY2^T^ strain produced any antibiotic compounds during laboratory culture. The strain was grown in a liquid culture medium for 7 days and organic extraction of the liquid culture was performed to create a crude metabolite extract. The extract was a bright cherry red in colour. The bioactivity of this metabolite extract was assessed against a panel of clinically relevant pathogenic bacteria by measuring the ZOIs (Fig. 6; for a full list of strains tested see Methods). Antibacterial activity was observed against Gram-positive strains including a clinical isolate of vancomycin-resistant and meticillin-resistant *Staphylococcus aureus* designated strain Mu50 [63] (ZOI diameter=10.3 mm) and the fusidic acid-/rifampicin-resistant *Enterococcus faecalis* JH2-2 [64] (ZOI diameter=12.2 mm). The antibiotic activity of the culture extract was somewhat dependent on the culture conditions used; for example, the antibiotic activity was drastically reduced in extracts using mannitol as the carbon source (Fig. S3). Further work is now ongoing to determine the extract component responsible for this bioactivity.

## Discussion

The results presented here outline the discovery of a novel species of *Streptomyces* and represent the first *Streptomyces* strain isolated in our wider efforts to isolate deep-sea sponge-associated microbes [30]. This study provides evidence that continuing to isolate actinomycetes, even those mined extensively such as *Streptomyces *, can lead to the discovery of taxonomic novelty and biosynthetic diversity. This supports prior large-scale global sequence analyses demonstrating that *Streptomyces *, in particular *Streptomyces* _RG1, not only have the highest number of GCFs among bacteria, but also have the highest number of yet uncharacterized and unknown GCFs [24]. The current study indicates that continuing to isolate, sequence and screen novel species within this group could represent an important route to the discovery of new natural products.

Our study suggests that GCF content is shaped both by vertical inheritance and LGT; strains share much of their GCF repertoire with close relatives, but also acquire individual GCFs from more distant lineages. Evidence on the extent and rate of LGT within *Streptomyces* is mixed. A phylogenetic study of LGT within *Streptomyces* suggested that only 10 LGT events occur every million years and that while the transfer of biosynthetic genes was overrepresented in the data, the transfer of entire intact BGCs was relatively rare [65]. In contrast, a study by Martinet *et al*. investigating 18 strains from the same species (*Streptomyces lunaelactis)* found that 54 % of BGCs were actually strain-specific [66]. This lack of conserved BGCs within a species highlights a limitation of this analysis, as different strains within the same species may have a variable biosynthetic repertoire, driven in part by the acquisition of new clusters from outside the clade. Additional aspects to consider are that fragmented GCFs will decrease the apparent vertical transmission, while the omission of GCFs present in only one or two strains will increase the apparent verticality. So, while it remains clear that phylogenetic distance plays a major role in the biosynthetic repertoire – with A15ISP2-DRY2^T^ sharing most of its GCFs with its closest relatives – exchange of BGCs over larger evolutionary distances is also important, as reflected in the six unique GCFs present in A15ISP2-DRY2^T^ but not found elsewhere in the clade (Table S8). This result is consistent with the view that lateral acquisition of BGCs is an important driver of biosynthetic diversity. In the future, a systematic study of *Streptomyces* radiation using these techniques might help to provide a global picture of the relative contributions of vertical descent and LGT to GCF content.

Here, we used BiG-SCAPE and ARTS to rapidly assess the biosynthetic novelty of A15ISP2-DRY2^T^ within its clade. Resistance-based mining or target-directed mining — the identification of resistance genes within BGCs — has led to the discovery of several first-in-class antibiotics from *Streptomyces* in recent years [57, 67]. ARTS identified that 19 of 34 BGC regions in A15ISP2-DRY2^T^ contained such potential resistance or a duplicated core gene, including two GCFs found exclusively in A15ISP2-DRY2^T^. These clusters represent priority targets for compound isolation or, if not produced under standard laboratory conditions, heterologous expression [68, 69]. A15ISP2-DRY2^T^ shared the majority of the identified GCFs with its closest relatives (* S. liliiviolaceus* BH-SS-21^T^ and *'S. dioscori'* A217^T^), despite these strains being isolated from drastically different environments: '*S. dioscori'* from a yam plant and *S. liliiviolaceus* from soil [70, 71]. Interestingly, the related strains show distinct bioactivities, with *'S. dioscori*' A217 having activity solely against Gram-negative *K. pneumoniae* [71] and *S. liliiviolaceus* BH-SS-21^T^ reporting activity against the Gram-negative plant pathogen *Ralstonia solanacearum* [70]. This suggests that the metabolomic profile of A15ISP2-DRY2^T^ is distinct from its close relatives and highlights the poorly understood relationship between the genome and metabolome of a strain [72, 73]. It is possible that different environmental niches may select for strains with distinct metabolomes rather than distinct BGCs [73]. The distinct antibiotic spectrum displayed by our novel deep-sea strain, therefore, warrants further investigation, and the characterization of the specialized metabolite(s) responsible is now a priority. Overall, the *in silico* and *in vitro* results presented here suggest this novel deep-sea *Streptomyces* is an excellent candidate for further antibiotic bioprospecting.

### Description of '*Streptomyces ortus'* sp. nov.

The systematic name proposed for the isolated strain A15ISP2-DRY2^T^ is '*Streptomyces ortus'* sp. nov. The species nomenclature *ortus* (or-tus), sunrise or risen, L. masculine. adj. reflects the deep-sea origins of the original isolate and the red colour that colonies display when grown on ISP2 media.

The type strain is A15ISP2-DRY2^T^ (NCIMB 15405^T^ = DSM 113116^T^), isolated from a deep-sea demosponge sponge identified as *Polymastia corticata* from the Gramberg Seamount in the Atlantic Ocean (depth 1869 m; latitude 15° 26' 43″ N ; longitude 51° 05' 46″ W). The isolate is an aerobic, Gram-positive, filamentous actinomycetota that forms branched substrate hyphae and aerial mycelia with spores (~0.82×1.27 µm) on mannitol soy flour agar. The strain has a high salt tolerance with growth up to 8 % (w/v) (1.37 M) NaCl. It was successfully cultured at pH 5–12 and at temperatures of 4, 15, 20, 28 and 37 °C. The strain had six complete rRNAs with each 16S rRNA gene sequence deposited in GenBank under accession numbers ON356021–ON356026. The genome size of the type strain is 9.27 Mb (GenBank accession no. JAIFZO000000000) and its genomic DNA G+C content is 70.83 mol%.

## Supplementary Data

Supplementary material 1
