# Supplementary material for: Discovery and biosynthetic assessment of 'Streptomyces ortus' sp. nov. isolated from a deep-sea sponge
Source: Microb Genom. 2023 May 11;9(5):mgen000996. doi: 10.1099/mgen.0.000996 (PMC10272871; doi:10.1099/mgen.0.000996)
Supplement: Supplementary material 1 [file mgen-9-996-s001.pdf]

Discovery and biosynthetic assessment of *Streptomyces ortus* sp nov. isolated from a deep-sea sponge

Supplementary material:

Table S1. Type strains of Streptomyces aurantiacus clade used in this study. NCBI GenBank accession numbers listed, BGC identified with antiSMASH 6.0, contigs listed not including scaffolds

***Streptomyces* species**	**Assembly Accession**	**BGCs**	**Contigs**	**Assembly size (mb)**
Streptomyces umbrinus JCM 4521^T	*BMUM00000000*	41	71	11.86
Streptomyces glomeroaurantiacus JCM 4677^T	AP023440	28	1	9.43
'Streptomyces dioscori' A217	*PYBJ00000000*	43	57	10.24
Streptomyces tauricus JCM 4837^T	*BMVY00000000*	39	107	11.04
Streptomyces liliifuscus sp. nov. ZYC-3^T	CP066831	30	2	11.03
Streptomyces liliiviolaceus sp. nov. BH-SS-21^T	JAGPYQ000000000	39	4	11.13
Streptomyces fructofermentans JCM 4956^T	BMWD00000000	26	116	8.87
Streptomyces albicerus TRM68295^T	VWMY00000000	48	294	11.97
Streptomyces apricus SUN51^T	VDFC00000000	43	692	8.81
Streptomyces ortus sp. nov. A15^T	JAIFZO000000000	34	9	9.29

Table S2. Summary statistics for the genome assembly of strain A15ISP2-DRY2^T. Assembled using Unicycler and scaffolded with MeDuSa, assembly metrics assessed with QUAST (contigs >= 500bp), Bowtie2 and Qualimap2.

Assembly metric	Assembly
**Number of contigs**	9
**Number of scaffolds**	4
**Scaffolds >= 1000bp**	2
**Total length (Mb)**	9.29
**Largest contig (Mb)**	5.23
**Largest scaffold (Mb)**	8.61
**N50 (Mb)**	8.61
**L50**	1
**GC content**	70.83%
**Mapped reads**	98.72%
**Error rate**	0.8%
**Coverage**	77.6391

Table S3. Top 10 BLASTN (2.13.0+) hits for the deep-sea sponge 700bp COI gene sequence. NCBI accession: OP036683

Description	Scientific Name	Query Cover	% Identity
Polymastia corticata mitochondrial partial COI gene for cytochrome oxidase subunit 1	*Polymastia corticata*	92%	**100%**
Polymastia littoralis mitochondrion, complete genome	*Polymastia littoralis*	99%	**95.76%**
Polymastia atlantica voucher TS2947 cytochrome c oxidase subunit I (COX1) gene	*Polymastia atlantica*	94%	**96.31%**
Polymastia atlantica voucher TS2938 cytochrome c oxidase subunit I (COX1) gene	*Polymastia atlantica*	94%	**96.31%**
Polymastia sp. 2 PRT-2020 voucher TS3976 cytochrome c oxidase subunit I (COX1) gene	*Polymastia sp. 2 PRT-2020*	94%	**96.02%**
Sphaerotylus strobilis voucher TS3628 cytochrome c oxidase subunit I (COX1) gene	*Sphaerotylus strobilis*	94%	**95.87%**
Sphaerotylus strobilis voucher TS4700 cytochrome c oxidase subunit I (COX1) gene	*Sphaerotylus strobilis*	94%	**95.87%**
Sphaerotylus strobilis voucher TS4699 cytochrome c oxidase subunit I (COX1) gene	*Sphaerotylus strobilis*	94%	**95.87%**

Description	Scientific Name	Query Cover	% Identity
Sphaerotylus strobilis voucher TS4697 cytochrome c oxidase subunit I (COX1) gene	*Sphaerotylus strobilis*	94%	**95.87%**
Sphaerotylus strobilis voucher TS2685 cytochrome c oxidase subunit I (COX1) gene	*Sphaerotylus strobilis*	94%	**95.87%**

Table S4. NCBI Prokaroyate genome annotation pipeline results for genome

Annotation Provider	NCBI
Annotation Date	10/21/2022 13:51:25
Annotation Pipeline	NCBI (PGAP)
Annotation Method	Best-placed reference protein set; GeneMarkS-2+
Annotation Software revision	6.3
Features Annotated	Gene; CDS; rRNA; tRNA; ncRNA; repeat_region
Genes (total)	8,130
CDSs (total)	8,043
Genes (coding)	7,794
CDSs (with protein)	7,794
Genes (RNA)	87
rRNAs	6, 6, 6 (5S, 16S, 23S)
complete rRNAs	6, 6, 6 (5S, 16S, 23S)
tRNAs	66
ncRNAs	3
Pseudo Genes (total)	259
CDSs (without protein)	259
Pseudo Genes (ambiguous residues)	0 of 259
Pseudo Genes (frameshifted)	80 of 259
Pseudo Genes (incomplete)	193 of 259
Pseudo Genes (internal stop)	19 of 259
Pseudo Genes (multiple problems)	40 of 259
CRISPR Arrays	1

Table S5. Table of single-copy orthologous genes present in the A15ISP2-DRY2^T genome assembly as expected for a genome from the order streptomycetales (lineage dataset: streptomycetales_odb10).

**Assembler**	**Complete [Single copy/Duplicated]**	**Fragmentated**	**Missing**	**Number of BUSCO groups searched**
**Unicycler**	99.7% [99.4%/0.3%]	0.1%	0.2%	1579

Table S6. GGDC formula 2 (d4) dDDH (TGYS) and ANI values for 10 closely related strains to A15ISP2-DRY2^T and M. echinospora ATCC 15837 used as an outgroup

**Strain**	**dDDH (d4, in %)**	**C.I. (d4, in %)**	**G+C % difference**	**FASTANI (%)**
Streptomyces liliiviolaceus BH-SS-21	45.8	[43.3 - 48.4]	0.04	93.3053
Streptomyces dioscori A217	45.1	[42.6 - 47.7]	0.11	93.0804
Streptomyces tauricus JCM 4837	43.7	[41.2 - 46.3]	0	92.7929
Streptomyces glomeroaurantiacus JCM 4677	35.2	[32.8 - 37.7]	0.54	89.7836
Streptomyces apricus SUN51	34.7	[32.3 - 37.2]	1.27	89.4996
Streptomyces liliifuscus ZYC-3	31.6	[29.2 - 34.1]	0.61	87.8961
Streptomyces umbrinus JCM 4521	31.6	[29.2 - 34.1]	0.66	87.8979
Streptomyces albicerus TRM68295	31.5	[29.1 - 34.0]	0.81	87.5189
Streptomyces ederensis JCM 4958	31.5	[29.1 - 34.0]	0.46	86.239
Streptomyces fructofermentans JCM 4956	29.2	[26.8 - 31.7]	1.58	83.6057
Streptomyces stelliscabiei DSM 41803	25.1	[22.8 - 27.6]	0.27	83.554
Streptomyces caniscabiei NE06-02D	25	[22.7 - 27.5]	0.45	83.5369
Micromonospora echinospora ATCC 15837	18.9	[16.7 - 21.2]	1.54	75.9895

Table S7. antiSMASH results with closest ClusterBlast hit 6.1.1 and antiSMASH db 3.0

Region	Type	ClusterBlast Hit antiSMASHdb	Most Similar Known Cluster	Similarity
**1**	NRPS, T3PKS	Streptomyces dioscori strain A217 (100%)	Herboxidiene	10%
**2**	Terpene	Streptomyces dioscori strain A217 (100%)	2-methylisoborneol	100%
**3**	NRPS	Streptomyces albireticuli strain MDJK11 (23%)	Foxicins A-D	29%
**4**	Siderophore	Streptomyces dioscori strain A217 (87%)	*No similar cluster*	
**5**	NAPAA	Streptomyces dioscori strain A217 (58%)	Rapamycin	17%
**6**	Ecotine	Streptomyces dioscori strain A217 (100%)	Ecotine	100%
**7**	Terpene	Streptomyces dioscori strain A217 (85%)	Albaflavenone	100%
**8**	PKS-like, T1PKS	Streptomyces coelicolor A3(2) CFB_NBC_0001 (70%)	Arsono-polyketide	91%
**9**	T3PKS	Streptomyces sp. CS131 (30%)	Alkylresorcinol	66%
**10**	NRPS, T3PKS, terpene	Streptomyces aquilus strain GGCR-6 (37%)	Feglymycin	68%
**11**	T2PKS, Ladderane	Streptomyces sp. 3214.6 (53%)	Simocyclinone D8	40%
**12**	Terpene	Streptomyces dioscori strain A217 (40%)	Isorenieratene	100%
**13**	NRPS	Streptomyces dioscori strain A217 (36%)	Borrelidin	4%
**14**	NRPS	Streptomyces dioscori strain A217 (89%)	Rimosamide	21%
**15**	NRPS	Streptomyces dioscori strain A217 (56%)	Diisonitrile antibiotic SF2768	55%
**16**	Lanthipeptide- class-iii, RiPP-like	Streptomyces sp. S1A1-3 (100%)	Informatipeptin	42%

**17**	T1PKS, terpene	Streptomyces sp. VN1 (81%)	Oxalomycin B	9%
**18**	Terpene	Streptomyces dioscori strain A217 (60%)	Herboxidiene	4%
**19**	NRPS, NRPS-like, T1PKS, other, terpene	Streptomyces griseochromogenes strain ATCC 145 (49%)	Aurantimycin A	48%
**20**	Terpene	Streptomyces dioscori strain A217 (100%)	Hopene	92%
**21**	NRPS-like, PKS-like, T1PKS, ecotine	Streptomyces dioscori strain A217 (65%)	Showdomycin	17%
**22**	Siderophore	Streptomyces dioscori strain A217 (100%)	Grincamycin	8%
**23**	NAPAA	Streptomyces bottropensis ATCC 25435 (63%)	Stenothricin	13%
**24**	Terpene	Streptomyces dioscori strain A217 (63%)	Geosmin	100%
**25**	RiPP-like	Streptomyces sp. YIM 130001 DSC45 s06 (80%)	*No similar cluster*	
**26**	NRPS, NRPS-like, betalactone	Amycolatopsis alba DSM 44262 (42%)	Vazabitide A	23%
**27**	Lanthipeptide class iv	Streptomyces yanglinensis strain CGMCC 4.2023 (25%)	*No similar cluster*	
**28**	Siderphore	Streptomyces dioscori strain A217 (100%)	*No similar cluster*	
**29**	PKS-like, RRE-containing, T2PKS	Streptomyces dioscori strain A217 (84%)	Cinerubin B	100%
**30**	Melanin	Streptomyces dioscori strain A217 (75%)	Melanin	60%
**31**	Siderophore	Streptomyces dioscori strain A217 (100%)	Desferrioxamin B/E	83%
**32**	RiPP-like	Streptomyces geranii strain A301 (88%)	*No similar cluster*	
**33**	Nucleoside	Streptomyces dioscori strain A217 (66%)	*No similar cluster*	

**34**	NRPS	Streptomyces sp. 150FB (15%)	Lysolipin I	4%

Table S8: BiG-FAM database information of GCF singletons found in S. ortus. Total does not include putative members.

**BGC Class**	**Most similar known cluster**	**BiG-FAM family**	**Comment on taxonomy of BiG-FAM family**	**Core member?**
Siderophore	No similar cluster	*GCF_01140*	*Staphlococcus 62.9%. Streptomyces 2.8% (6391 total)*	*TRUE*
NRPS	*NRPS Foxicins A-D 29%*	*GCF_09952*	*Rhodococcus with 7 found in streptomyces (22 total)*	*FALSE*
Terpene	*Isorenieratene 100%*	*GCF_00998*	*72% Streptomyces (211 total)*	*FALSE*
T2PKS, Ladderane	*Simocyclinone D8 40%*	*GCF_03444*	*Rare only 2 BGCs in GCF. All streptomyces*	*FALSE*
NRPS	*Lysolipin I 4%*	*GCF_00012*	*Mainly found in Pseudomonas – (25972 total)*	*FALSE*
NRPS,T3PKS, Terpene	*Feglymycin 73%*	*GCF_00057*	*Nonomuraea 2 core members 15 putative (2 total)*	*FALSE*

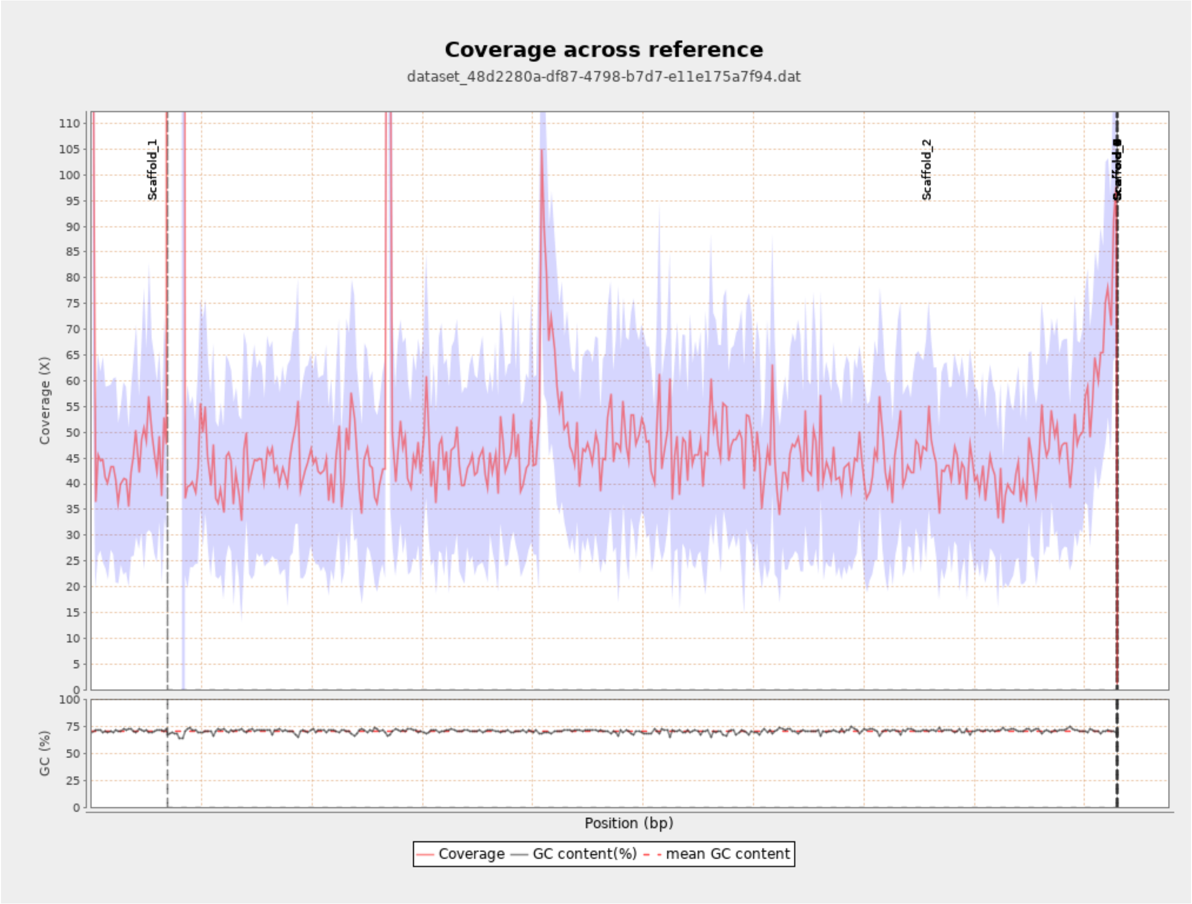

Figure S1: Qualimap2 figure from Bowtie2 alignment of Illumina reads against the final assembly. Coverage (top) and GC % (bottom) across the final assembly.

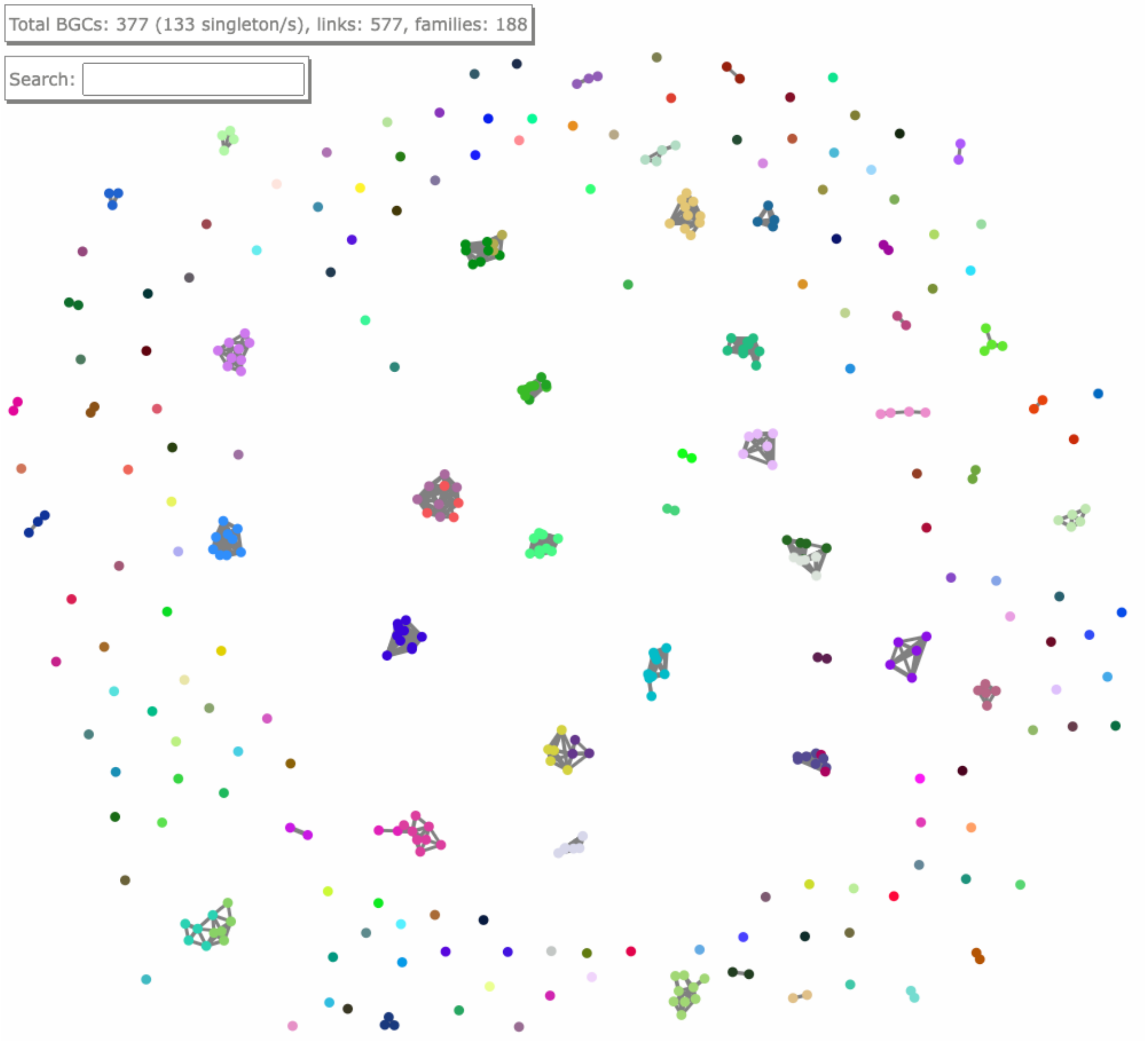

Figure S2. Full BiG-SCAPE (v1.1) GCF family network from the S. auranticus clade including singletons. GCF clustering cutoff 0.35. Direct screen capture from index.html file.

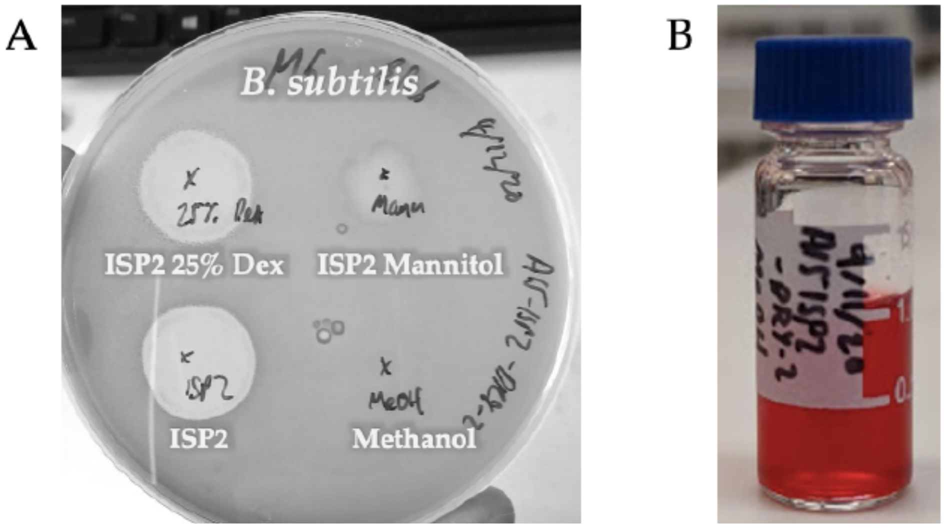

Figure S3. A) Bioactivity of 10µL crude extract on soft agar containing B. subtilis. The activity was reduced when the strain was grown with mannitol instead of dextrose, but activity was unaffected if dextrose concentration was reduced to 1g/L (25% of standard). B) The culture extract was a cherry red colour
